# A Paper on Opium in Certain Forms of Fever, and in Diarrhœa and Dysentery

**Published:** 1866-02

**Authors:** S. B. Hawley

**Affiliations:** Aurora, Ill.


ARTICLE V.

### A PAPER ON OPIUM IN CERTAIN FORMS OF FEVER, AND IN DIARRHŒA AND DYSENTERY.

BY S. B. HAWLEY, M.D., of Aurora, Ill.

Read before the Aurora City Medical Association, Dec. 1st, 1865.

My own experience has led me to believe that opium exercises a pernicious effect in certain forms or conditions of fever, and in a large number of cases of chronic diarrhœa and dysentery. The conditions of fever in which it has seemed to me to be a hurtful drug, are those denominated typhoid, and, particularly, that stage of fever marked by dryness of the mouth and fauces, constant thirst, and nervous, trembling motions of the muscular system. I am convinced that to give opium, or any of its preparations, when this condition exists, will increase the difficulty and prolong the time before convalescence.

This conviction rests upon personal observation of cases treated side by side, and consecutively with and without the use of opiates; and, if my observations are not at fault, even the single sleeping powder given at bedtime delays the process of cure.

In chronic diarrhœa and dysentery, when the patient exhibits a tongue but slightly furred, characterized, rather, by a pale look and elongated state of the papillæ, with edges of the tongue red, has constant thirst, though the mouth may not appear dry when we see him, and some feeling as if bloated, I have never seen any, to me, satisfactory result follow the use of opiates.

In the summer of 1863, while I was on duty with my regiment, the 35th Ill., near Murfreesboro, Tenn., the dysentery prevailed extensively in our camps, and opium still held a prominent place in my mind as a suitable remedy in that disease. I shall, therefore, relate a case that occurred under my treatment and observation that season, and which is but one of very many similar cases that I could relate, bearing on the point I wish to elucidate:—

Private M., of Co. H., 35th Ill., applied to me for relief, on account of dysentery, about April 3d. I prescribed oleum ricini, ℥ss, tr. opii, ʒj, at once, and pulvis Doveri, grs. xx, ft. ch. No. iv., one to be taken each night and morning, and excuse from duty two days, at the end of which time he went on duty, thinking his trouble cured. But, in about two weeks, he reported, as bad as at the first. I then directed him to have:—

℞. Pill Hy., ------------------------------ grs. x.
Opii, ------------------------------ grs. vj.
Qui. S., ------------------------------ grs. v.

Ft. Pills No. viij. To take one every four hours, and moved off with oleum ricini.

The second day after, I put him upon pills of quinine and opium, āā grs. j., to take one such three times a-day, for three days, and then grain pills of opium twice a-day for several days. This treatment produced considerable relief, so that he again went on duty for a time, but still reported at sick call every few days, saying that he was not quite cured.

In May, Dr. T., who had been for many months on duty in the hospitals of Rolla and Louisville, joined the regiment, and I asked him to take charge of M.'s case. He did so, and prescribed with some seeming temporary benefit, but still the disease ran on. I observed his prescriptions far enough to know that opium formed a principal part of most of them.

About one month later, Dr. J. D. W. came on duty with the regiment, and I placed M. under his care, as he was still declining in general strength, and having four or five dysenteric discharges each day. He remained under his care till about the middle of July, taking opium and astringents, more or less, most of the time.

Up to this time, he had declined steadily, and then presented the following symptoms:—tongue covered with whitish, swollen papillæ, the posterior half a yellowish-white, smooth fur, the front showing the papillæ distinctly; he had little appetite; tumid abdomen; constant thirst, though the mouth appeared not very dry upon inspection. He was able to walk about the camp, and had about half a dozen stools a-day, one or two like a watery diarrhœa, and the balance mucus with an occasional tinge of blood.

He had often been carefully questioned, as to any chill, and had taken considerable quinine, under the belief that malaria was keeping up the disease; and some relief had seemed to result from time to time from the use of this remedy, but not lasting, and no chill had been clearly made out to exist. I, therefore, determined to try a, to me, new treatment in his case, though I confess that at the time he seemed too much reduced for the course of treatment which the event proved quite *apropos* to his case. I gave him sulph. magnesia, ℥ss., tartar emetic, grs. ss., quinine S. grs. v. at once, about 8 o'clock A.M. At 1 o'clock P.M., it not having moved the bowels at all freely, I repeated the dose which produced copious stools. That night he reported a more comfortable night's rest than he had had for three months, and for two days he had a better appetite, and there was some cleaning of the tongue; the papillæ approached their natural color; the bloat began to subside; and he showed a more cheerful aspect.

On the third day, there was some return of the old symptoms when I repeated the above prescription, and I gave directions to repeat it, less the tartar emetic, every other day for ten days. From this period he steadily convalesced, and was able to go through the terrible work of Chickamauga and Chattanooga that fall.

Many cases might be added to the point of laying aside opium and all astringents in these cases, but I shall proceed to show the rationale of my conclusions:—

"Opium," says Dr. WOOD, "is a stimulant narcotic. Taken by a healthy person, in a moderate dose, it increases the force, fulness, and frequency of the pulse, augments the temperature of the skin, invigorates the muscular system, quickens the senses, animates the spirits, and gives new energy to the intellectual faculties. Its operation, while thus extending to all parts of the system, is directed with peculiar force to the brain, the functions of which it excites sometimes even to intoxications or delirium. In a short time this excitation subsides, a calmness of the corporeal functions succeeds, and at the end of half an hour or hour from the administration of the narcotic all consciousness is lost in sleep. The soporific effect having continued for eight or ten hours, goes off, and is generally succeeded by more or less *nausea*, *headache*, *tremors, and other symptoms of diminished or irregular nervous action.* All secretions, with the exception of that from the skin, are either suspended or diminished, and the peristaltic motion of the bowels is lessened. When larger doses are taken, the period of excitement and exhilaration is shorter, the soporific and anodyne effects are more intense and of longer duration, and the succeeding symptoms of debility are more obvious and alarming."

The strong language he uses, in setting forth these final effects of opium, is almost identical with that used by STILLE, PEREIRA, CHRISTISON, ORFILA, and COPELAND in the same connection, and was, I think, intended to put us on our guard against its use in many cases where we have been accustomed to think it harmless, if not useful. The great comfort which an anodyne brings to a nervous patient, suffering moderate pain, with uneasy and sympathetic nurses, has often, I think, led us to the use of this drug, when we had no good therapeutic reason for so doing, and, also, when there existed the best of reasons why we should not give it.

In typhoid, typhus, and bilious fevers, the secretions are usu ally nearly all locked up, as shown by the dry tongue, continued thirst, and diminished flow of urine, while water drank in the usual mode is not absorbed, as is natural, by the stomach, but flows on through the bowels and passes off in a diarrhœa. Why give a medicine that lessens the peristaltic action and dries up all the secretions, save that from the skin, when the disease produces the above set of symptoms? Do we believe in *similia similibus curantur* in these diseases? Or can we, as the Dover's powder propose, blow hot and cold in the same breath? I doubt it.

I would not be understood to urge the entire abandoning of opiates in these diseases, for, at times, we will find patients suffering under a degree of pain that must be relieved before anything else can be done. But I believe if we could discover the absolute status of these cases, we should find that every moment we continued the use of opiates in these diseases beyond the time required to relieve unendurable pain, prolongs the time before convalescence. And this, I believe, I have shown upon philosophical grounds, that is, that the opiate naturally increases the very condition we have to meet.

Likewise, in chronic diarrhœa and dysentery, when a dry mouth, constant thirst, and scanty urine, shows the natural secretions all deficient, it seems to me that opium, or any astringent, is most unphilosophical, though they may lessen the number of stools. What element of our systems does opium supply, that shall in any degree compensate for the fact that it produces nausea, headache, tremors, and other symptoms of nervous debility, while it dries up all the secretions? Are there not many and good reasons for believing that the torpor which it produces stops the very process by which nature is at work to remove the cause of the disease.

I make no attempt to exhaust this topic, but hope to place clearly before your minds this, to me, glaring inconsistency of practice which has so long prevailed among physicians.

